# Identification of 3 subpopulations of tumor-infiltrating immune cells for malignant transformation of low-grade glioma

**DOI:** 10.1186/s12935-019-0972-1

**Published:** 2019-10-11

**Authors:** Jiacheng Lu, Hailin Li, Zhengxin Chen, Ligang Fan, Shuang Feng, Xiaomin Cai, Huibo Wang

**Affiliations:** Department of Neurosurgery, First Affiliated Hospital of Nanjing Medical University, Nanjing, 210029 Jiangsu China

**Keywords:** Glioma, Tumor immune, Malignant transformation, Prognosis

## Abstract

**Background:**

Tumor-infiltrating immune cells (TIICs) are highly relevant to clinical outcome of glioma. However, previous studies cannot account for the diverse functions that make up the immune response in malignant transformation (MT) from low-grade glioma (LGG) to high-grade glioma (HGG).

**Methods:**

Transcriptome level, genomic profiles and its relationship with clinical practice were obtained from TCGA and CGGA database. The “Cell type Identification By Estimating Relative Subsets Of RNA Transcripts (CIBERSORT)” algorithm was used to estimate the fraction of 22 immune cell types. We divided the TCGA and CGGA set into an experiment set (n = 174) and a validation set (n = 74) by random number table method. Univariate and multivariate analyses were performed to evaluate the 22 TIICs’ value for MT in LGG. ROC curve was plotted to calculate area under curve (AUC) and cut-off value.

**Results:**

Heterogeneity between TIICs exists in both intra- and inter-groups. Several TIICs are notably associated with tumor grade, molecular subtypes and survival. T follicular helper (TFH) cells, activated NK Cells and M0 macrophages were screened out to be independent predictors for MT in LGG and formed an immune risk score (IRS) (AUC = 0.732, p < 0.001, 95% CI 0.657–0.808 cut-off value = 0.191). In addition, the IRS model was validated by validation group, Immunohistochemistry (IHC) and functional enrichment analyses.

**Conclusions:**

The proposed IRS model provides promising novel signatures for predicting MT from LGG to HGG and may bring a better design of glioma immunotherapy studies in years to come.

## Background

Gliomas account for 60% of all primary and other central nervous system (CNS) tumor diagnoses, and make up ~ 80% of all malignant brain tumors [1]. The World Health Organization (WHO) classifies gliomas according to histology and molecular subtype, and grades them by the scale of I, II, III, IV. low-grade gliomas (LGG) typically range from grades I–II, while high-grade gliomas (HGG) are categorized as grades III–IV. Glioblastoma multiforme (GBM) is grade IV glioma subtype which often spontaneously appears in the CNS, but can also progress from LGG. GBM takes up half of CNS tumors, and is a fatal disease with no curable therapy [2]. Even with a comprehensive therapy, such as surgical resection, adjuvant radiotherapy, and alkylating agent temozolomide chemotherapy, patients who suffer from gliomas still have short median survival time, due to the aggressiveness of tumors, resistance to treatments, and recurrence over time [3]. In particular, patients with GBM approximately has a median survival of only 14–16 months [4]. In the past decade, studies on the anticancer immune responses for other tumors have promoted clinical advances in the limited success of conventional therapies. Meanwhile, the discovery of CNS lymphatic system has provided a new theoretical basis and opportunity for brain tumor immunotherapy [5].

Tumor-infiltrating immune cells (TIICs), whose function and composition subtly altered with the immune status of the host have been reported to be effectively targeted by drugs correlate with clinical outcome [6]. Melanoma and non-small-cell lung cancer are the two solid tumors in which immunotherapy has proved to be effective [7]. However, compared with these two tumors, glioma harbors a lower burden of somatic mutations and a more immunosuppressive tumor microenvironment [8]. Unique challenges should be overcome before immunotherapy applied to CNS. First, anatomically, the blood–brain barrier (BBB) restricts the entry of immune cells to the brain parenchyma. Also, the tumor cells themselves secrete a variety of immunosuppressive factors that influence macrophage polarization, dendritic cell (DC) maturation, regulatory T cell recruitment, inhibition of neutrophil and natural killer (NK) cell function. Previous studies have revealed that glioblastomas are heavily infiltrated with monocytes/microglia, although TIICs are relatively rare. Reports suggest that these cells account for 10–30% of viable cells within the tumor mass. They appear to be affected by tumors and have positive immunosuppressive effects. For example, Rodrigues et al. demonstrated that normal monocytes that come into contact with glioblastoma cells secrete multiple immunosuppressive factors (IF-10, TGF-β, B7-H1), have reduced phagocytic ability and induce apoptosis in activated T cells [9]. While preclinical data shows the success of immunotherapy for gliomas, the profiles of TIICs in glioma and their clinical value still remain to be explained.

Nevertheless, Immunohistochemistry and flow cytometry are the two most commonly used techniques that depend on a single marker for detecting TIICs in previous studies. Obviously, these approaches can be misleading and are not comprehensive as many marker proteins are expressed in different cell types. The “Cell type Identification By Estimating Relative Subsets Of RNA Transcripts” (CIBERSORT) employs deconvolution of bulk gene expression data and a sophisticate algorithm for in silico quantification of many immune cell types in heterogeneous samples as tumor stroma. Here, we used CIBERSORT, for the first time, to quantify the 22 TIICs subpopulations of immune response in glioma based on the patients’ gene expression profiling from TCGA and CGGA public databases in order to investigate its relationship between clinicals factors, with the final goal of developing new immunotherapeutic strategies.

## Materials and methods

### Datasets

**Fig. 1:**
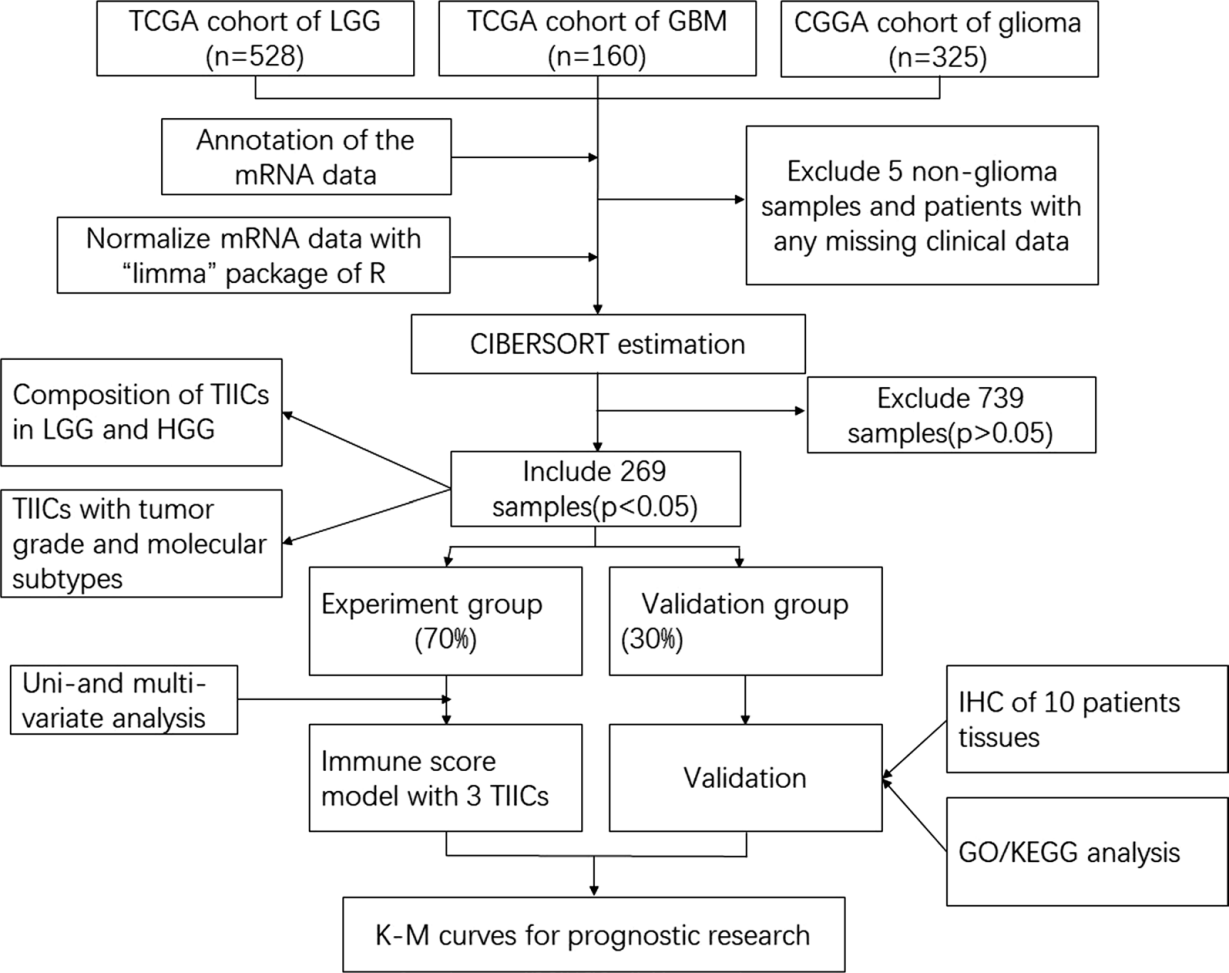
Details of the study design. *TCGA* The Cancer Genome Atlas, *CGGA* Chinese Glioma Genome Atlas, *CIBERSORT* Cell type Identification By Estimating Relative Subsets Of RNA Transcripts

We examined expression data and clinical variables from the following main sources: The Cancer Genome Atlas (TCGA) dataset (http://cancergenome.nih.gov/),Chinese Glioma Genome Atlas (CGGA) dataset (http://www.cgga.org.cn) (up to April 10, 2019). We searched the supplements and contacted the investigators to get the missing information for samples. In TCGA dataset, we obtained mRNAseq data of 160 GBM samples and 528 LGG samples. 325 samples generated by Illumina HiSeq platform were collected form CGGA dataset, ranging from WHO grade II to grade IV. Then, expression profiles of each samples and corresponding clinical data were manually organized. Besides, 5 non-GBM patient data from the TCGA-GBM dataset, patients with any missing or insufficient data on age or survival data were excluded from subsequent processing. RNA sequencing data were firstly transformed using “voom” (variance modeling at the observational level) for the two datasets. Details of the study design are illustrated in Fig. 1 as a flowchart.

### CIBERSORT estimation

The gene expression with standard annotation were uploaded to the CIBERSORT web portal (http://cibersort.stanford.edu/), and the algorithm was running the LM22 signature and 1000 permutations. Cases with a CIBERSORT output of p < 0.05, indicating that the inferred fractions of TIICs populations produced by CIBERSORT are accurate, were considered to be eligible for further analysis. For each sample, the final CIBERSORT output estimates were normalized to sum up to one and thus can be interpreted directly as cell fractions for comparison across different immune cell types and dataset.

### Immunohistochemical detection of immune cell types

5 LGG and 5GBM tissue from 10 patients who received surgery in the First Affiliated Hospital of Nanjing Medical University (Nanjing, Jiangsu province, China) were constructed for immunohistochemistry. Specimens were all confirmed by pathological analysis as glioma. IHC was performed as described earlier, using monoclonal antibodies against CXCR5, CD4, CD68, CD11b, CD57 and CD56 (H-132; Santa Cruz Biotechnology, Santa Cruz, CA). Isotype-matched mouse monoclonal antibodies were used as negative controls. Slides were analyzed using an image analysis workstation (Spot Browser, ALPHELYS). Polychromatic high-resolution spot-images (740 × 540 pixel, 1.181 μm/pixel resolution) were obtained (200× fold magnification). The density was recorded as the number of positive cells per unit tissue surface area. For each duplicate, the mean density was used for statistical analysis.

### Gene oncology (GO) and Kyoto encyclopedia of genes and genomes (KEGG)

GO was applied to determine the function of differentially expressed genes and pathway enrichment was analyzed by KEGG (http://string-db.com).

### Statistical analyses

Statistical analyses were conducted using R software version 3.5.3 (http://www.r-project.org/) and SPSS 19.0 for windows (IBM, NY, USA). All statistical tests were two-sided and a *p* value < 0.05 is considered as significant.

Hierarchical clustering of immune cell proportions was conducted to compare distinct immune cell infiltration in different samples. The proportions of various TIICs were defined as a change from 0 to 1 based on our observation. R packages “Corrplot” (https://github.com/taiyun/corrplo), “Pheatmap” (https://CRAN.R-project.org/package=pheatmap) and “Vioplot” (https://github.com/TomKellyGenetics/vioplot)were also used to investigate differences in the composition of immune cells within and between groups. Wilcoxon test was used to evaluate the relationship between tumor grades, tumor molecular subtypes and TIICs. The association between TIICs and survival were analyzed using log rank test and Kaplan–Meier (K–M) curve visualized the results. Multivariable analyses were further operated to screen independently predictors. AUC and cut-off value were obtained by conducting ROC curve. “Limma” package was used to analysis the differential expressed gene, |log2FC| > 1.3219 and FDR < 0.05 were set as filters.

## Results

### Composition of immune cells in LGG and GBM

**Fig. 2:**
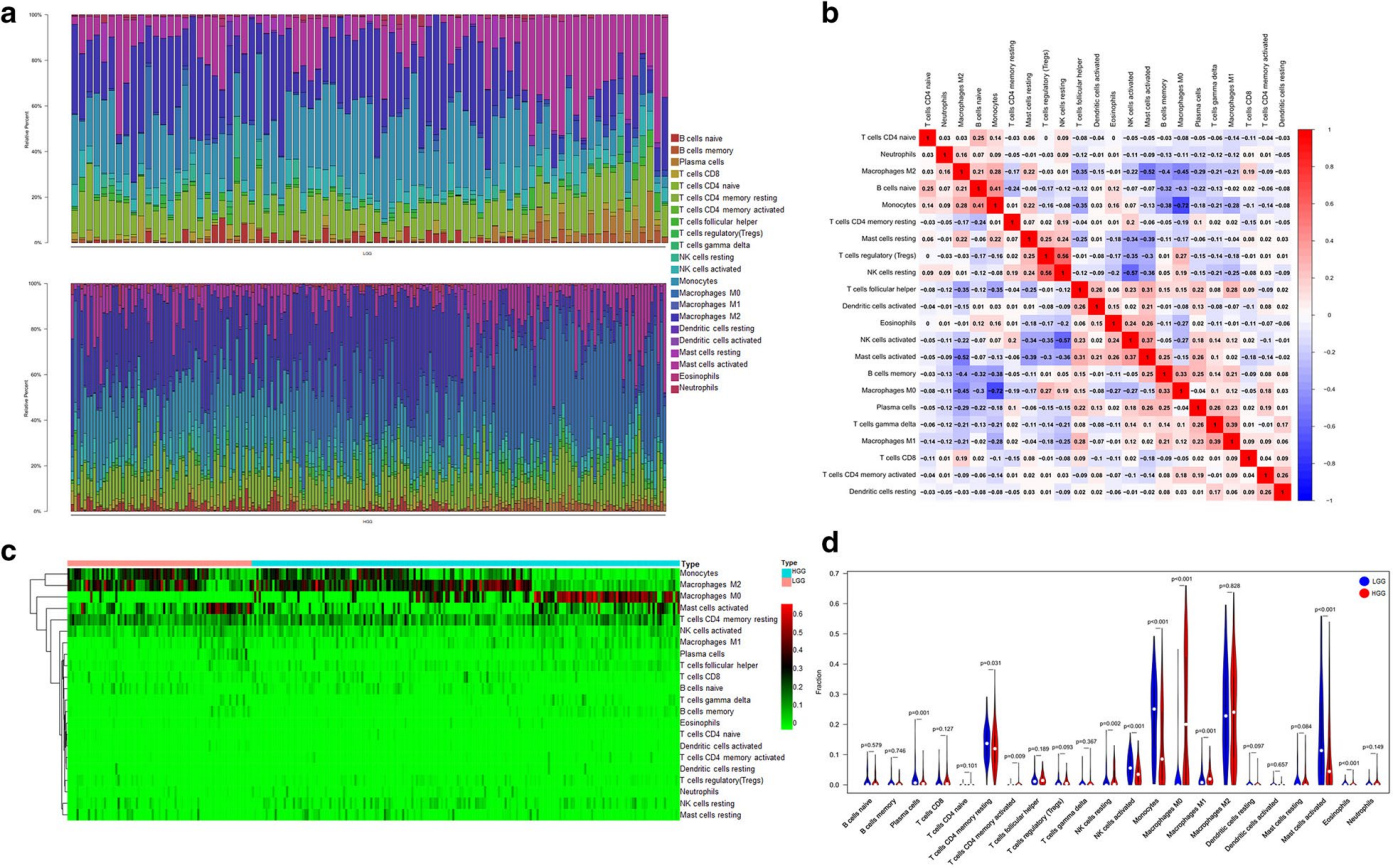
The landscape of immune infiltration in glioma. **a** The difference of immune infiltration between low- and high-grade glioma. **b** Correlation matrix of all 22 immune cell proportions in TCGA and CGGA datasets. **c** Heatmap of 22 immune cell proportions. The horizontal axis shows the clustering information of samples which were divided into two major clusters. **d** The distribution of same immune cells between low- and high-grade glioma. p-values show the significance of the distribution

“Limma” package [10] run firstly to normalize the gene expression data and to accommodate the operational requirements of CIBERSORT. Then, CIBERSORT algorithm was used to analysis the difference of immune infiltration between LGG and HGG samples in 22 subpopulations of immune cells. 269 out of the total 1008 samples from TCGA and CGGA datasets with p-value < 0.05 were included for subsequent processing, of which 81 samples were grouped into LGG cohort and 188 samples into the HGG cohort. The total value of all immune cells in each sample was set at one, Fig. 2a showed the proportion of all 22 subpopulations of immune cells in these samples (Fig. 2a). Obviously, the proportions of immune cells in glioma varied significantly between both intra- and inter-group. Resting NK cells and T cells regulatory (Tregs) exhibited a significant positive correlation, while there was a distinctive negative correlation between M0 macrophages and monocytes by average linkage clustering (Fig. 2b). Through hierarchical clustering based on the above data, we can find that TIICs, such as monocytes, M0 macrophages showed striking distribution differences in LGG and HGG (Fig. 2c). The violin plot (Fig. 2d) showed that there were marked differences in the distribution of 10 out of 22 immune cells, such as monocytes (p < 0.001), M0 macrophages (p < 0.001), activated NK cells (p < 0.01), between LGG and HGG cohorts. Taken together, these results suggest that the heterogeneity of TIICs in gliomas is evident and may play a role in the malignant progression of LGG to HGG.

### Immune cells associated with tumor grade and molecular subtypes

**Fig. 3:**
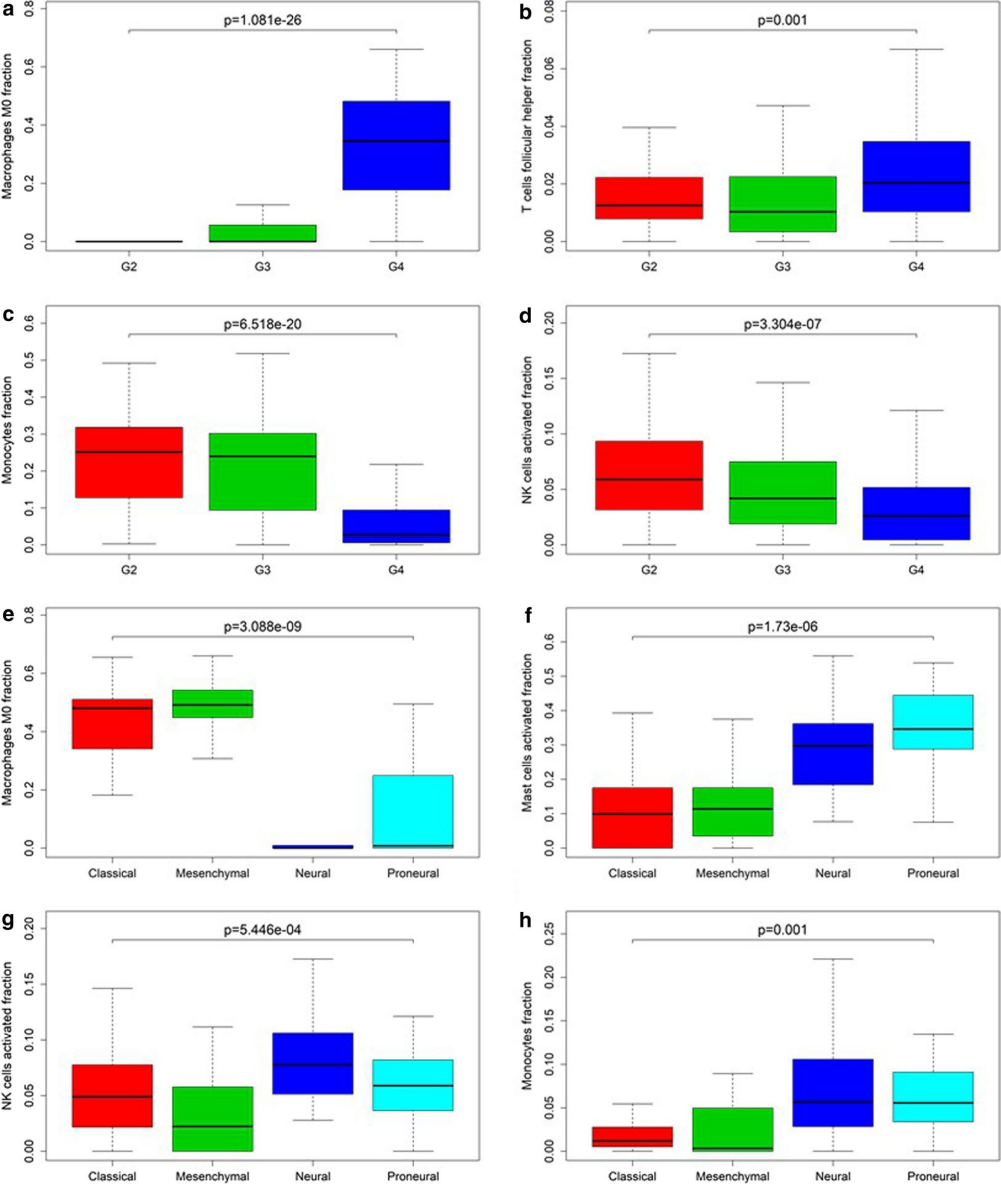
Immune cells associated with tumor grade and molecular subtypes. **a**–**d** Relationship between the fraction of M0 macrophages (p < 0.001), TFH cells (p = 0.001), monocytes (p < 0.001) and activated NK cells (p < 0.001) and glioma WHO grade. **e**–**h** Relationship between the fraction of M0 macrophages (p < 0.001), activated Mast cells (p < 0.001), activated NK cells (p < 0.001) and monocytes (p = 0.001) and molecular subtypes of glioma

Wilcoxon tests were used to evaluated the relationship between tumor grades, tumor molecular subtypes and TIICs. p-values are shown in Table 1. TIICs such as M0 Macrophages and TFH cells are positively correlated with elevated levels (Fig. 3a, b), while cell subpopulations like monocytes and activated NK cells were negatively correlated (Fig. 3c, d). Subsequently, due to the lack of molecular subtypes information in TCGA dataset, we analyzed the relationship between glioma molecular subtypes and TIICs in samples from CGGA dataset. p-values are also shown in Table 1. Differences exist in the distribution of tumor-infiltrating cells of various glioma molecular subtypes (Fig. 3e–h). These results further demonstrate that TIICs may affect the progression of glioma to some extent.

**Table 1:** Comparison of CIBERSORT immune cells fractions between grade and molecular subtypes of glioma

TIICs	Grade	Molecular subtype
	p-value	p-value
B cells naive	*2.81E*−*05*	0.188257963
B cells memory	*4.61E*−*05*	0.737169605
Plasma cells	*0.001815233*	*3.55E*−*05*
T cells CD8	0.249592172	0.172157201
T cells CD4 naive	*0.039037853*	0.413131088
T cells CD4 memory resting	*0.002288205*	0.146932516
T cells CD4 memory activated	0.014882526	0.171750522
T cells follicular helper	*0.001064575*	0.091842131
T cells regulatory (Tregs)	0.074373931	*4.73E*−*05*
T cells gamma delta	0.564203378	*0.002882824*
NK cells resting	*0.00140453*	*0.000258713*
NK cells activated	*3.30E*−*07*	*0.000544631*
Monocytes	*6.52E*−*20*	*0.001213765*
Macrophages M0	*1.08E*−*26*	*3.09E*−*09*
Macrophages M1	*0.000436372*	0.377244738
Macrophages M2	0.078628638	0.730798047
Dendritic cells resting	0.094204212	*0.001100304*
Dendritic cells activated	0.364620103	0.158098129
Mast cells resting	*0.035430526*	*0.008667249*
Mast cells activated	*0.000954643*	*1.73E*−*06*
Eosinophils	*3.56E*−*05*	0.058457676
Neutrophils	*0.019156303*	*0.047975933*

### Immune cell comparison responding to the prognosis of gliomas

**Fig. 4:**
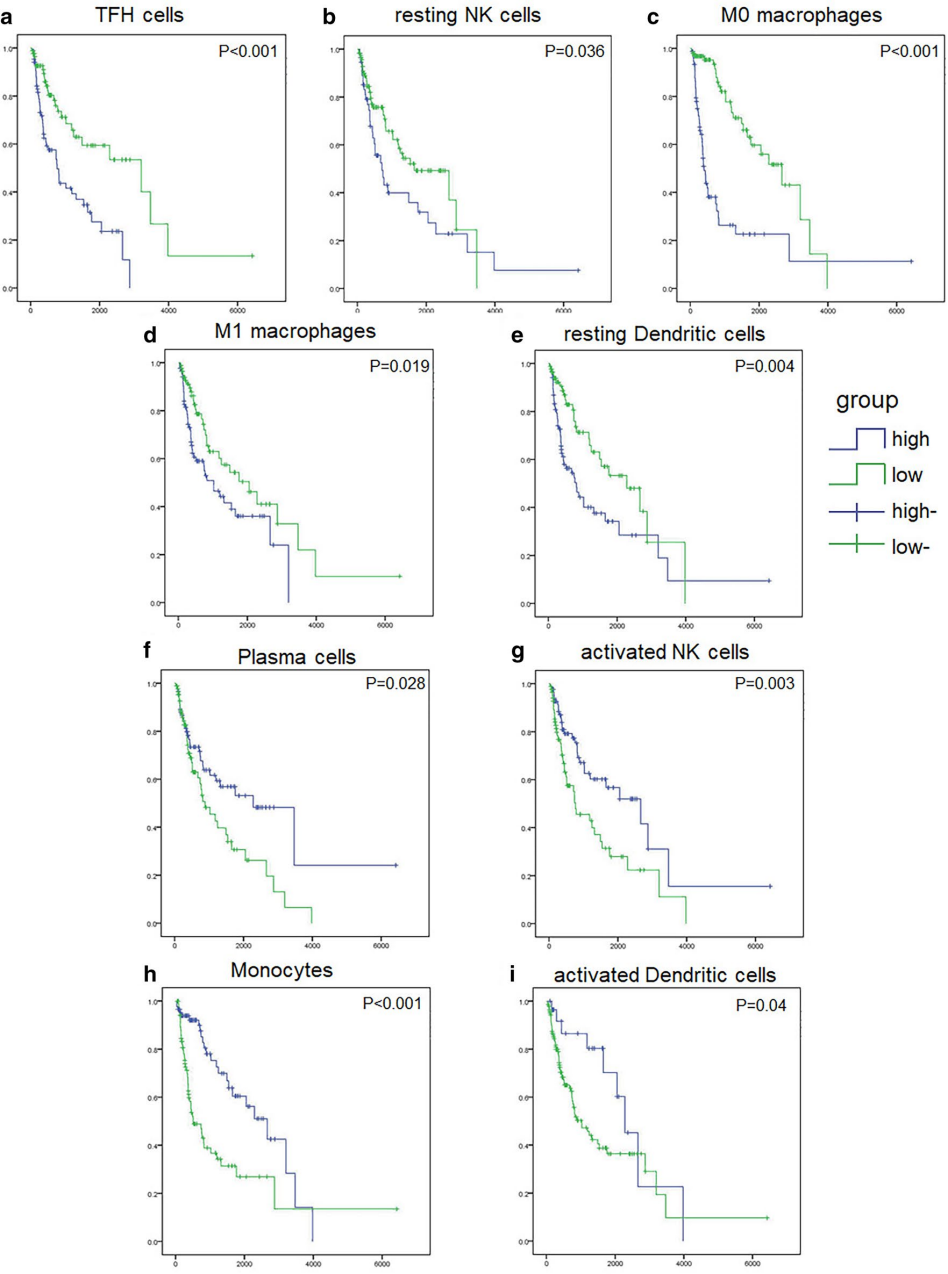
Nine subpopulations of TIICs significantly associated with the prognosis of patients with glioma in the experiment group. **a**–**e** Kaplan–Meier plots visualized high level of immune cells, including TFH cells (p < 0.001), resting NK cells (p = 0.036), M0 macrophages (p < 0.001), M1 macrophages (p = 0.019) and resting dendritic cells (p = 0.004), associated with poor OS. **f**–**i** Kaplan–Meier plots visualized high level of immune cells, including plasma cells (p = 0.028, activated NK cells (p = 0.003), monocytes (p < 0.001) and activated dendritic cells (p = 0.04), associated with good OS

We then obtained clinical data from TCGA and CGGA databases, samples with a survival time less than 30 days were excluded. Then expression profiles of each samples and corresponding clinical data were manually organized. To further analyze the factors associated with patient prognosis and perform subsequent validation, we randomly divided the total sample into 70% of the experiment group and 30% of the validation group. For immune cell infiltration and corresponding survival time, we performed univariate analyses (Table 2) and nine immune cells showed significant prognostic value in both databases. Highly expressed T follicular helper cells, resting NK cells, M0 macrophages, M1 macrophages and resting Dendritic cells predicted poor overall survival, while high expression of the other 4 cell subpopulations, including plasma cells, activated NK cells, monocytes and activated dendritic cells predicted a better prognostic value. Kaplan–Meier curves visualizes the above results (Fig. 4a–i). The consequences of the univariate analyses further sought out that these nine TIICs specifically influence patients’ prognosis and paved the way for further screening independent predictors.

**Table 2:** Univariate and multivariate analysis between 22 subpopulations of TIICs and survival in experiment group

TIICs	Univariate analysis			Multivariate analysis			
	HR	p-value	95% CI	HR	B	p-value	95% CI
B cells naive	0.709	0.158	0.440–1.143				
B cells memory	0.068	1.558	0.968–2.507				
Plasma cells	0.594	0.028*	0.371–0.949	0	− 7.889	0.181	0–39.380
T cells CD8	1.071	0.778	0.664–1.730				
T cells CD4 naive	0.229	0.144	0.032–1.652				
T cells CD4 memory resting	0.736	0.208	0.456–1.187				
T cells CD4 memory activated	1.875	0.121	0.848–4.145				
*T cells follicular helper*	2.641	*<* 0.001*	1.585–4.400	1.69E+06	14.339	0.046*	1.298–2.196E+12
T cells regulatory (Tregs)	0.979	0.929	0.613–1.563				
T cells gamma delta	1.304	0.354	0.743–2.289				
NK cells resting	1.653	0.036*	1.027–2.660	0	− 9.67	0.099	0–6.150
*NK cells activated*	0.498	0.003*	0.310–0.800	0	− 22.69	*<* 0.001*	0–Inf
Monocytes	0.367	*<* 0.001*	0.223–0.603	0.141	− 1.959	0.259	0.005–4.228
*Macrophages M0*	4.103	*<* 0.001*	2.500–6.733	10.527	2.354	0.018*	1.501–73.813
Macrophages M1	1.763	0.019*	1.089–2.852	2157.986	7.677	0.072	0.503–9.261E+6
Macrophages M2	0.768	0.27	0.481–1.227				
Dendritic cells resting	2.017	0.004*	1.243–3.274	0	− 8.748	0.423	0–3.094E+5
Dendritic cells activated	0.486	0.04*	0.241–0.983	0	− 62.875	0.233	0–3.495E+17
Mast cells resting	0.928	0.821	0.486–1.774				
Mast cells activated	0.76	0.248	0.477–1.210				
Eosinophils	0.678	0.106	0.424–1.086				
Neutrophils	1.03	0.904	0.641–1.655				

*Statistically significant

### Identify several independent prognostic factors

**Fig. 5:**
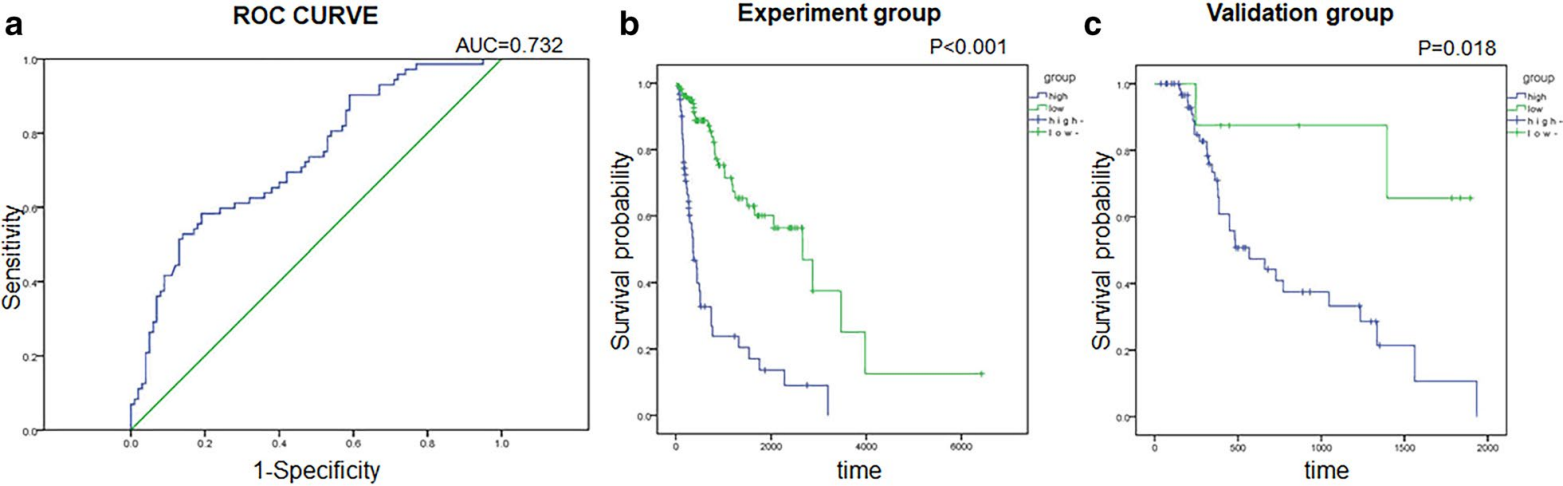
IRS construction and validation. **a** ROC curve of the IRS model in experiment group. **b** Kaplan–Meier curve visualized the overall survival of experiment group based of the level of IRS. **c** Kaplan–Meier curve visualized the overall survival of validation group based of the level of IRS

Multivariate analysis was conducted to identify independently prognostic factors and the p-values were also shown in Table 2. The result suggested that these three TIICs, including TFH cells, activated NK cells and M0 macrophages, may serve as independent predictors of the progression of glioma, especially in the MT of LGG to GBM. Based on the correlation coefficients obtained by multivariate analysis, we constructed an immune risk score (IRS) model based on these three immune cells: IRS = 14.339*TFH cells + 2.354* M0 macrophages − 22.69* activated NK cells. ROC curve was than drawn and we therefore got the AUC = 0.732 (Fig. 5a) and figured out cut-off value = − 0.43124. Next, we divided the experiment group into high- and low-risk groups by cut-off value after calculating IRS. K–M curve indicated a significant difference in survival between high- and low-risk groups (Fig. 5b). What’s more, IRS scores showed statistical differences with molecular subtypes of glioma (Table 3).

**Table 3:** Relationships between IRS scores and molecular subtypes of glioma

IRS	Subtype				p-value
	Classical	Mesenchymal	Neural	Pronerual	
Grade					
LGG	(0.18 ± 0.52)	(0.90 ± 0.64)	(0.24 ± 0.59)	(0.98 ± 0.81)	< 0.05
HGG	(0.86 ± 0.72)	(1.02 ± 0.48)	(− 0.87 ± 0.59)	(− 0.02 ± 0.65)	< 0.05

### Verify the prognostic model in the validation group

For the validation group, we calculated IRSs and also divided it into high- and low-risk groups based on the cut-off value. The K–M curve showed the survival rate of patients in the high-risk group was significantly worse than that in the low-risk group (Fig. 5c). This was a good proof of the validity of the IRS model we constructed.

### IHC confirmed the CIBERSORT result

**Fig. 6:**
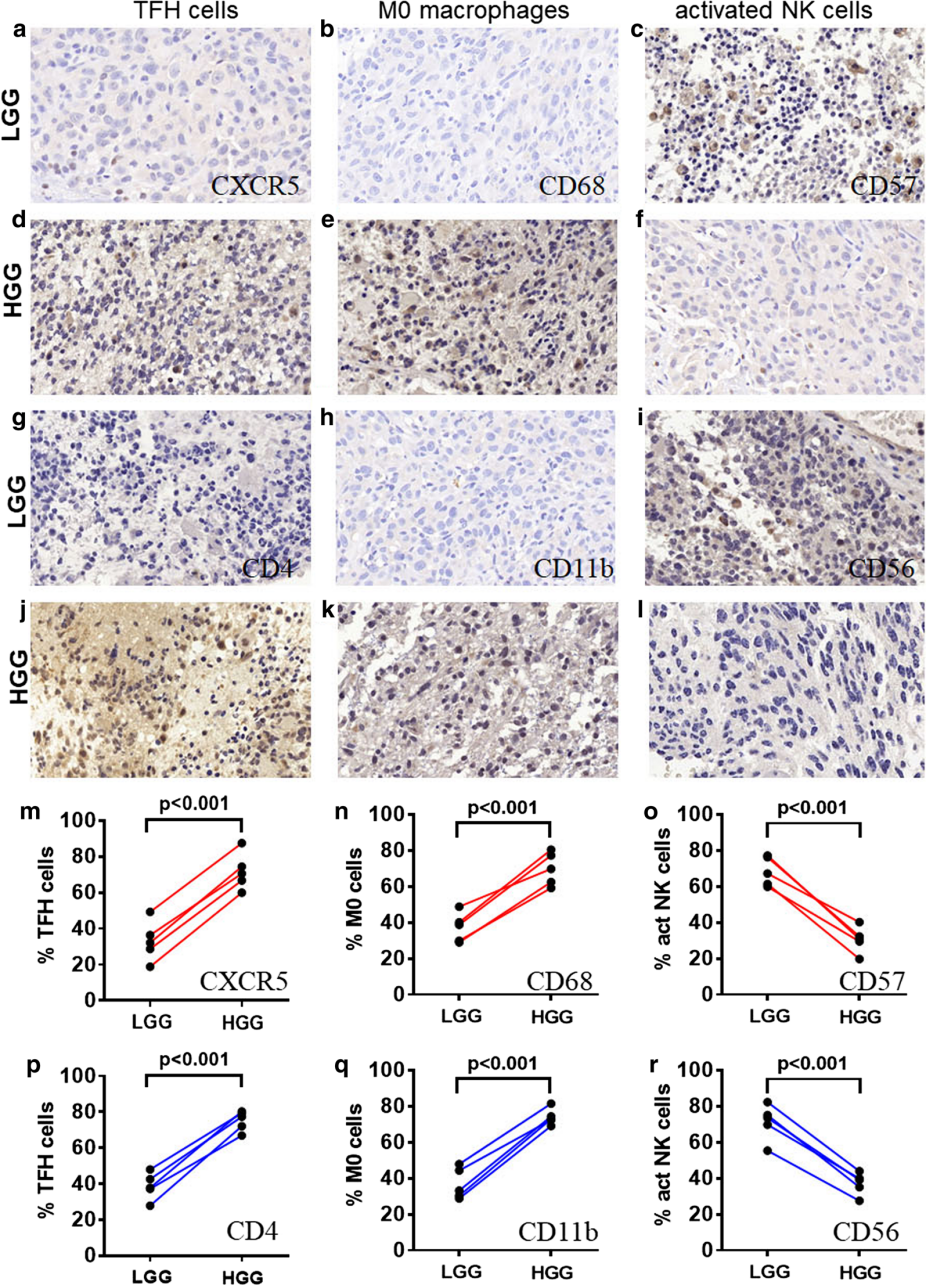
IHC of three significant TIICs. **a**–**c**, **g**–**i** Level of TFH cells, M0 macrophages and activated NK cells in LGG group. **d**–**f**, **j**–**l** Level of TFH cells, M0 macrophages and activated NK cells in HGG group. **m**–**r** The quantification of TFH cells (p < 0.001), M0 macrophages (p < 0.001) and activated NK cells (p < 0.001) are shown

In order to verify the explorative data obtained for TFH cells, activated NK cells and M0 macrophages, we evaluated these cells density by immunohistochemistry in 5 human LGG tumor tissues and 5 human GBM tumor tissues Examples of these cells tryptase staining and quantification summary are shown in Fig. 6a–r. In agreement with CIBERSORT results, activated NK cells was reduced in GBM while TFH cells and M0 macrophages were increased in GBM.

### Differential expressed genes and enrichment analysis based on prognostic model

**Fig. 7:**
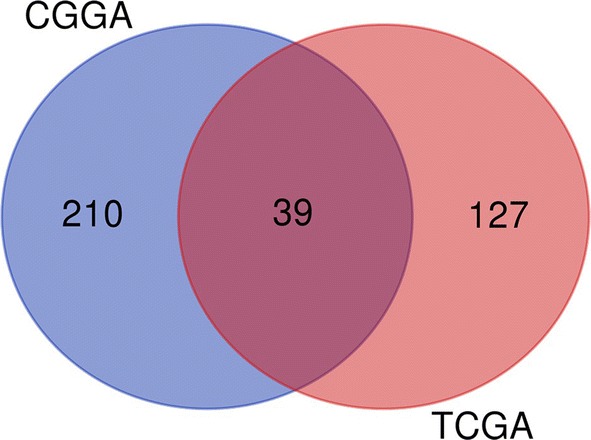
Venn diagram of the differential expressed genes. 249 DEGs from CGGA datasets and 166 DEGs from TCGA were taken to obtain the 39 common DEGs

We calculated IRSs for all samples and divided them into high- and low-risk group for TCGA and CGGA databases. After obtaining the differential genes of the two databases separately, we found that we got 118 common differential expressed genes for the intersection of the results (Fig. 7). For these genes, online tools “STRING” was used for GO/KEGG enrichment analysis and found that they enriched in the following biological processes (Table 4) which were mainly related to immune response.

**Table 4:** GO enrichment and KEGG pathway analysis results with grouped all samples into high- and low-IRS group

GO/KEGG	Pathway ID	Pathway description	False discovery rate
GOBP	GO.0022617	Extracellular matrix disassembly	2.24E−05
	GO.0030198	Extracellular matrix organization	5.75E−05
	GO.0040012	Regulation of locomotion	7.51E−05
	GO.0030334	Regulation of cell migration	0.0002
	GO.0001503	Ossification	0.000601
GOCC	GO.0005615	Extracellular space	1.16E−10
	GO.0044421	Extracellular region part	3.19E−06
	GO.0005576	Extracellular region	9.92E−06
	GO.0005578	Proteinaceous extracellular matrix	0.000868
	GO.0044420	Extracellular matrix component	0.000868
	GO.0005793	Endoplasmic reticulum-Golgi intermediate compartment	0.00291
GOMF	GO.0005518	Collagen binding	0.00543
	GO.0031730	CCR5 chemokine receptor binding	0.0436
KEGG	4512	ECM-receptor interaction	0.000163
	4151	PI3K-Akt signaling pathway	0.00375
	4510	Focal adhesion	0.00375
	910	Nitrogen metabolism	0.0339

## Discussion

For a long time, although it is well known that immune cells play an important role in tumor initiation and development, these insights have few influence on clinical practice [11, 12]. In addition, the role of genes that are abnormally expressed in tumor tissues in diagnosis and prognosis has also attracted widespread attention; however, few studies have focused on the differential distribution of immune cells between different components. In this paper, we firstly established an immune risk score model based on the fractions of three subpopulations of TIICs. Compared with the high-IRS group based on our model, the low-IRS group has a significantly better survival rate (p < 0.001). This finding suggests that our IRS model can better predict progression of glioma, especially in the MT from LGG to GBM. Validation group, IHC and functional enrichment analyses further illustrate the validity of the model. This study opens a door for a better understanding of new diagnosis strategy from the perspective of TIICs. We acknowledge that there exist limitations in this research, particularly no precise analysis of the effect of single TIICs. Besides, studies on TIICs in initiation of glioma weren’t carried on due to lack of sequencing samples from normal people in these public databases. Therefore, further studies are urgently needed to analysis single TIISs and whether it is possible to detect the real-time progression of tumor through the state of immune cells in the circulatory system.

Gliomas are tumors of CNS, originating from transformed neural stem or progenitor glial cells [13]. On the basis of histopathological characteristics WHO classified gliomas into groups: low-grade gliomas (LGG, grades I and II) are well differentiated, slow-growing tumors, whereas high-grade gliomas (HGG, grades III and IV) are less differentiated or anaplastic, and strongly infiltrate brain parenchyma [14]. Glioblastoma (GBM) is categorized as the most malignant type (grade IV). It accounts for 50% of CNS tumors and is a deadly disease without curable therapy. Despite aggressive treatments, such as extensive resection combined with radiation and/or chemotherapy, patients with GBM eventually die of their disease [4]. In another aspect, patients with LGG may survive for many years, but after transformed to GBM, survival rates rapidly decline [15, 16]. A population-based study showed that the mean period of malignant transformation from LGG to GBM was 5.3 years and for anaplastic astrocytoma to GBM was 1.4 years [17]. Most of the predictive models established in previous studies on glioma development and malignant transformation were based on differential expressed genes, but they neglected that immune cells may also play an important role in tumorigenesis. Due to technical limitations, previous researches were limiting to a narrow insight of tumor-infiltrating cells. Immunohistochemistry and flow cytometry which depend on a single surface marker were used to evaluated TIICs. Apparently, these techniques may have misidentified other cell with the same surface markers as TIICs and are subjectively affected by observers. Thus, in the current study, we employed a silicon analysis, known as CIBERSORT, to infer the proportions of 22 immune cell subpopulations from glioma transcriptomes. CIBERSORT is a deconvolution algorithm for charactering TIICs composition of complex tissues by analyzing 547 gene expression, introduced by Newman etc. in 2015. They firstly employed a novel application of liner support vector regression to deconvolve the tissue composition. In order to assess the feasibility of TIICs deconvolution from bulk tumors, they then designed and validated a TIICs gene signature matrix, termed LM22. By using LM22 to deconvolve 3061 human transcriptomes, they therefore proved CIBERSORT has great specificity and sensitivity [11]. As an emerging technology, CIBERSORT have already conducted in breast cancer [18], lung cancer [19], colon cancer [6] and so on, all these studies demonstrated the effectiveness and accuracy of this tool when analyzing TIICs.

Univariate and multivariate analyses indicated TFH cells, activated NK cells and M0 macrophages as independent predictors. Then, based on their correlation coefficients, we firstly constructed such an IRS model in glioma. Among these correlation coefficients, or the degree to which the cell distribution correlated with tumor progression, the coefficient of activated NK cells is negative, while the coefficients of the other two TIICs are positive. This is consistent with our previous analyses between TIICs and tumor grade. Hence, we have adequate reason to believe that this model can predict MT between LGG and GBM well.

Immune system can be functionally divided into innate immunity and adaptive immunity, where adaptive immunity is antigen-specific. It mainly consists of B cell-mediated humoral immunity and cytotoxic T cell-mediated cellular immune responses, and both these two adaptive immunity processes require signals from CD4 T cells [9]. In one aspect, some CD4 T cell subpopulations such as Th1 cells can exert anti-tumor immunity by overcoming the tolerance of autoantibodies expressed by tumors, and these effectors T cells are advantageous for tumor immunotherapy [20]. However, other subsets of CD4 T cells, particularly regulatory T cells and TFH cells, inhibit tumor immunity, thereby promoting cancer growth [21–23]. In our study, although there was no significant difference in the composition of TFH cells between LGG group and HGG group, but in Fig. 2b we can see that its level in the GBM group is higher than that of the lower grade gliomas. At present, there is no research on the role of TFH in the immune microenvironment of glioma, which is the problem we need to think about and solve next.

Unlike T cells, NK cells play a unique role in innate and adaptive immune responses without the involvement of major histocompatibility complex (MHC) antigens or antibodies [24], and monitor status of intracellular bacteria, viruses-infected cells and transformed cells. Activated NK cells are one of two types of lymphokine-activated killer (LAK) cells. When stimulated by IL-2, they become activated against tumor cells. Although no randomized controlled trails of immunotherapy with HGG by LAK has been performed to date, one study showed patients treated with LAK cells had longer survivals than control groups [25]. Due to the difficulties in producing sufficient LAK cells, researches on activated NK cells for immunotherapy of glioma have been restricted. We pointed out a significant difference in the distribution of activated NK cells between low- and high-grade gliomas (p < 0.001) (Fig. 2c, d), and the lower the level of activated NK cells in the higher grade of gliomas (p < 0.001) (Fig. 3d). In studies of the association with glioma molecular subtype, the level of activated NK cells was the lowest in the mesenchymal subtype, which has the worst prognosis, while the other three subtypes harbor relatively higher level of it (p < 0.001) (Fig. 3g). In addition, as previously stated, the correlation coefficient of activated NK cells is also negative. These results indicate that activated NK cells may induce favorable clinical outcome of glioma, in another word, it may also be a vital suppressor for MT in LGG.

TAMs are macrophages infiltrating in tumor tissues which are the main composition in tumor microenvironment (TME). They differentiate through alternative pathways, among which the most common one is Notch pathway [26, 27]. What’s more, they facilitate tumor progression [28]. Once activated, monocytes continue to differentiate, first differentiated into M0 macrophages and then M1 and M2 arisen from M0. Others have shown that increased level of M0 is associated with poor clinical outcomes of lung adenocarcinoma [29]. So far, no clear experiments have been conducted to demonstrate the relationship between TAMs and glioma prognosis. Some people believe that TAMs in gliomas may be affected by tumor tissues and show immunosuppressive effects [19]. According to our work, the contents of M0 (p < 0.001) in GBM is higher than that of LGG. Wilcoxon test result also exhibits a gradual increase in the level of M0 from LGG to GBM. Moreover, coefficient of our IRS model also indicate that M0 comes under the influence of tumor development and promote malignant progression.

To again insight into the immune-related biological processes during glioma progression, we performed GO biological process (GOBP), GO cellular component (GOCC), GO molecular function (GOMF) and KEGG pathway analysis. Not only do the top results are immune-related, it is particularly worth mentioning that the first of each analysis are all immunologically relevant. This proves the validity of our consequences to some extent, on the other hand, it also finds some hub pathways in the MT of glioma, which indicates a path for future researches.

## Conclusion

In summary, our study expounded the distinct composition of tumor-infiltrating immune cells in different grades and molecular subtypes of glioma. The complex intersection between TIICs and MT was quantified by our IRS model. Finally, we pointed out some relevant pathways related to progression and MT of glioma. These findings deepen the understanding of immune responses in CNS tumors and may enable to develop more effective immunotherapeutic strategies.

## Abbreviations

TIICs: tumor-infiltrating immune cells
MT: malignant transformation
LGG: low-grade glioma
HGG: high-grade glioma
CIBERSORT: Cell type Identification By Estimating Relative Subsets Of RNA Transcripts
AUC: area under curve
TFH: T follicular helper
IHC: immunohistochemistry
CNS: central nervous system
WHO: World Health Organization
GBM: glioblastoma multiforme
DC: dendritic cell
NK: natural killer
TCGA: The Cancer Genome Atlas
CGGA: Chinese Glioma Genome Atlas
GO: gene oncology
KEGG: Kyoto encyclopedia of genes and genomes
IRS: immune risk score
K–M: Kaplan–Meier
MHC: major histocompatibility complex
LAK: lymphokine-activated killer
TME: tumor microenvironment
